# Supplementary material for: Investigative power of Genomic Informational Field Theory (GIFT) relative to GWAS for genotype-phenotype mapping
Source: bioRxiv. 2024 Apr 16:2024.04.16.589524. Preprint. [Version 1] doi: 10.1101/2024.04.16.589524 (PMC11042307; doi:10.1101/2024.04.16.589524)
Supplement: Supplementary file 1 [file media-2.docx]

**List if significant SNPs (99% confidence interval) by GWAS (page 1)**

| **CHRM** | **NAME** | **POSITION** | **-**${\mathbf{Log}}_{\mathbf{10}}(\mathbf{p}\mathbf{GWAS})$ | ${\boldsymbol{N}}_{\boldsymbol{-}}$ | ${\boldsymbol{N}}_{\boldsymbol{+}}$ | ${\boldsymbol{N}}_{\boldsymbol{0}}$ |
| --- | --- | --- | --- | --- | --- | --- |
| 6 | OAR6_32516276 | 28676510 | 7.544967291 | 94 | 181 | 292 |
| 6 | s71628 | 29009593 | 6.246299843 | 92 | 217 | 258 |
| 6 | OAR6_33291928 | 29450053 | 6.101131284 | 443 | 10 | 114 |
| 6 | OAR6_40277406 | 36073405 | 9.572402573 | 89 | 202 | 275 |
| 6 | OAR6_40311379 | 36104954 | 9.537964864 | 0 | 292 | 275 |
| 6 | OAR6_40855809 | 36655091 | 9.863933586 | 76 | 229 | 262 |
| 6 | OAR6_40955920 | 36750972 | 7.100108288 | 72 | 238 | 257 |
| 6 | DU178311_404 | 36839547 | 8.668877525 | 244 | 56 | 267 |
| 6 | OAR6_41424992 | 37203894 | 6.241510426 | 17 | 362 | 186 |
| 6 | OAR6_41558126 | 37334387 | 7.186312219 | 301 | 47 | 218 |
| 6 | OAR6_42576838 | 38310652 | 7.304654762 | 78 | 220 | 269 |
| 6 | OAR6_45285663 | 40594243 | 6.708439356 | 132 | 146 | 289 |
| 6 | OAR6_45662944 | 40975597 | 6.074949854 | 377 | 10 | 180 |

**List if significant SNPs (99% confidence interval) by GIFT (pages 1 to 16)**

| **CHRM** | **SNP NAME** | **POSITION** | **-**${\mathbf{Log}}_{\mathbf{10}}(\mathbf{p}\mathbf{GIFT})$ | ${\boldsymbol{N}}_{\boldsymbol{-}}$ | ${\boldsymbol{N}}_{\boldsymbol{+}}$ | ${\boldsymbol{N}}_{\boldsymbol{0}}$ |
| --- | --- | --- | --- | --- | --- | --- |
| 0 | OAR1_5744254 | 0 | 16.43704186 | 202 | 87 | 278 |
| 0 | OAR15_46439840 | 0 | 14.49562683 | 10 | 447 | 110 |
| 0 | OAR4_41180788 | 0 | 16.3425955 | 411 | 13 | 143 |
| 0 | s12374 | 0 | 17.16859569 | 131 | 140 | 286 |
| 0 | s73955 | 0 | 14.31317442 | 306 | 44 | 217 |
| 0 | s13794 | 302 | 13.68659773 | 54 | 253 | 258 |
| 0 | s59474 | 4111 | 13.88124291 | 91 | 222 | 253 |
| 1 | s64747 | 748143 | 15.52919149 | 155 | 137 | 275 |
| 1 | s11992 | 4246644 | 14.98537532 | 161 | 125 | 275 |
| 1 | s50475 | 4254004 | 14.95140036 | 215 | 82 | 270 |
| 1 | OAR1_6722309 | 7192190 | 15.87294405 | 375 | 10 | 175 |
| 1 | OAR1_11691321 | 11982306 | 15.91988265 | 83 | 209 | 275 |
| 1 | OAR1_36268411 | 35305689 | 13.74109171 | 313 | 35 | 218 |
| 1 | s21447 | 35833194 | 14.85823124 | 96 | 235 | 235 |
| 1 | s30526 | 36443661 | 15.98630965 | 136 | 152 | 279 |
| 1 | OAR1_38526208 | 37481382 | 16.17967574 | 418 | 5 | 144 |
| 1 | OAR1_38551279 | 37504986 | 16.17967574 | 5 | 418 | 144 |
| 1 | OAR1_48599629 | 46874115 | 14.92922944 | 145 | 157 | 265 |
| 1 | OAR1_60333122 | 57316101 | 13.70065682 | 20 | 380 | 167 |
| 1 | OAR1_68555372 | 64813680 | 15.83467328 | 359 | 27 | 181 |
| 1 | OAR1_75586198 | 70731891 | 13.74862106 | 83 | 183 | 267 |
| 1 | OAR1_77147307 | 72165449 | 14.22021368 | 165 | 109 | 293 |
| 1 | OAR1_93645852 | 88167112 | 15.88612988 | 183 | 93 | 290 |
| 1 | OAR1_93664787 | 88186660 | 15.20563898 | 183 | 94 | 290 |
| 1 | s30877 | 88366446 | 16.19057687 | 299 | 36 | 230 |
| 1 | OAR1_95658466_X | 90001077 | 14.10663039 | 7 | 420 | 139 |
| 1 | OAR1_101890858 | 95617371 | 13.96274742 | 60 | 270 | 237 |
| 1 | s71483 | 100551565 | 14.8213266 | 31 | 321 | 215 |
| 1 | DU390992_212 | 101012030 | 16.03119204 | 472 | 4 | 91 |
| 1 | OAR1_110626692 | 102921077 | 13.9110239 | 289 | 53 | 225 |
| 1 | s74483 | 103658234 | 13.61048004 | 292 | 36 | 239 |
| 1 | s33396 | 103676649 | 13.58444382 | 383 | 19 | 161 |
| 1 | OAR1_117069007 | 108272813 | 14.00832543 | 225 | 74 | 267 |
| 1 | OAR1_122737941 | 113773533 | 16.99446154 | 58 | 271 | 238 |
| 1 | OAR1_122906056 | 113922339 | 15.51350495 | 59 | 267 | 237 |
| 1 | OAR1_122975063 | 113996479 | 15.3668881 | 19 | 380 | 168 |
| 1 | s65345 | 114824235 | 14.60304485 | 185 | 114 | 268 |
| 1 | s49384 | 117927713 | 13.83707902 | 49 | 269 | 249 |
| 1 | OAR1_127962239 | 118639747 | 15.24327511 | 446 | 7 | 114 |
| 1 | s61314 | 119188150 | 14.68368825 | 276 | 43 | 241 |
| 1 | OAR1_131707541 | 121557067 | 13.76531975 | 206 | 79 | 282 |
| 1 | OAR1_135563277 | 125050209 | 16.67668139 | 148 | 135 | 284 |
| 1 | OAR1_137004604 | 126428678 | 16.60922109 | 18 | 355 | 194 |
| 1 | OAR1_137125494 | 126532548 | 13.59435156 | 175 | 100 | 292 |
| 1 | OAR1_137481062 | 126860653 | 18.44971106 | 66 | 232 | 269 |
| 1 | OAR1_137567887 | 126940397 | 13.81387267 | 67 | 206 | 284 |
| 1 | OAR1_137752062 | 127123575 | 13.95088423 | 382 | 11 | 174 |
| 1 | s15832 | 127230834 | 14.56459141 | 254 | 59 | 254 |
| 1 | OAR1_137928314 | 127261069 | 16.27469264 | 55 | 271 | 241 |
| 1 | OAR1_142079316 | 131229251 | 13.72542722 | 390 | 12 | 165 |
| 1 | OAR1_143380398 | 132523540 | 15.53487599 | 144 | 124 | 299 |
| 1 | OAR1_144078987 | 133163948 | 14.00898964 | 310 | 22 | 235 |
| 1 | OAR1_144545940 | 133569458 | 17.73993185 | 110 | 160 | 297 |
| 1 | OAR1_148875501 | 137766059 | 14.05178061 | 20 | 365 | 182 |
| 1 | OAR1_167277333 | 155294701 | 17.66421667 | 108 | 182 | 277 |
| 1 | OAR1_170173310 | 157781844 | 13.61068271 | 106 | 161 | 267 |
| 1 | OAR1_185845001_X | 172443190 | 13.75349974 | 9 | 410 | 145 |
| 1 | OAR1_187054397 | 173614261 | 15.60043806 | 225 | 94 | 248 |
| 1 | OAR1_189570484 | 175871349 | 15.54900475 | 43 | 277 | 246 |
| 1 | s55687 | 177131981 | 18.36616197 | 301 | 31 | 235 |
| 1 | OAR1_191856774 | 177992978 | 13.64443625 | 163 | 90 | 280 |
| 1 | OAR1_208608268 | 193200871 | 14.17481784 | 355 | 24 | 188 |
| 1 | OAR1_219952416 | 203674456 | 16.55973913 | 57 | 236 | 273 |
| 1 | OAR1_225614564 | 208946860 | 17.51555273 | 52 | 281 | 234 |
| 1 | s59879 | 229542336 | 13.79723988 | 20 | 372 | 175 |
| 1 | OAR1_263896550 | 244356465 | 16.45318922 | 223 | 89 | 255 |
| 1 | OAR1_280355916 | 259083296 | 16.35933887 | 400 | 8 | 159 |
| 1 | OAR1_286058076 | 264487221 | 19.80036932 | 341 | 24 | 202 |
| 1 | OAR1_286759511 | 265054013 | 14.61295564 | 291 | 35 | 240 |
| 1 | s60732 | 268235697 | 13.57894953 | 188 | 104 | 275 |
| 2 | s26088 | 3208854 | 13.74041984 | 32 | 310 | 225 |
| 2 | OAR2_2049668 | 3367799 | 15.34947417 | 246 | 56 | 249 |
| 2 | OAR2_35966054 | 34658666 | 20.03977282 | 3 | 474 | 90 |
| 2 | OAR2_36546466 | 35201638 | 15.34927113 | 225 | 69 | 273 |
| 2 | OAR2_37954567 | 36544130 | 15.3539514 | 47 | 262 | 258 |
| 2 | s43405 | 38155780 | 16.45153483 | 29 | 354 | 183 |
| 2 | s72260 | 38334113 | 13.67214535 | 282 | 46 | 239 |
| 2 | OAR2_48363557 | 45552152 | 18.75555069 | 13 | 391 | 163 |
| 2 | OAR2_60262385 | 55908488 | 13.60190732 | 147 | 130 | 287 |
| 2 | s02535 | 56304104 | 13.63652895 | 231 | 65 | 271 |
| 2 | OAR2_61639386 | 57307599 | 13.82985875 | 277 | 46 | 244 |
| 2 | OAR2_64835451 | 60340361 | 14.58394514 | 55 | 279 | 233 |
| 2 | OAR2_73140784 | 68448937 | 15.37675757 | 393 | 18 | 153 |
| 2 | OAR2_77896643 | 73109758 | 15.20482434 | 452 | 6 | 109 |
| 2 | OAR2_82089332 | 76979186 | 13.93403492 | 20 | 388 | 159 |
| 2 | OAR2_94485428 | 88818925 | 15.59656308 | 303 | 51 | 212 |
| 2 | OAR2_94517955 | 88851122 | 16.20303738 | 41 | 319 | 207 |
| 2 | s06944 | 91063757 | 14.94351872 | 172 | 110 | 284 |
| 2 | OAR2_104831719 | 97338136 | 13.56442149 | 12 | 402 | 150 |
| 2 | OAR2_106512643 | 99013601 | 14.18112048 | 250 | 60 | 256 |
| 2 | OAR2_116423980 | 108264568 | 14.25161089 | 284 | 39 | 244 |
| 2 | OAR2_116898327 | 108750425 | 15.74504891 | 6 | 425 | 131 |
| 2 | OAR2_116920296 | 108775514 | 19.52881936 | 6 | 438 | 123 |
| 2 | OAR2_116938058 | 108780371 | 19.75180103 | 436 | 6 | 122 |
| 2 | s67620 | 112897395 | 15.67976337 | 356 | 19 | 192 |
| 2 | s13017 | 113364458 | 15.14398103 | 45 | 278 | 244 |
| 2 | OAR2_121369139 | 113449517 | 18.4260014 | 12 | 391 | 164 |
| 2 | OAR2_137337129 | 128972285 | 14.22979804 | 391 | 16 | 160 |
| 2 | s32561 | 131397735 | 13.84859226 | 114 | 171 | 282 |
| 2 | s28909 | 136485296 | 13.58580645 | 155 | 134 | 275 |
| 2 | OAR2_155018930 | 145994402 | 13.50332147 | 149 | 133 | 275 |
| 2 | OAR2_171247972 | 161641301 | 15.2507456 | 15 | 381 | 164 |
| 2 | OAR2_205458798 | 193912783 | 19.01572956 | 284 | 42 | 241 |
| 2 | OAR2_205643974 | 194084442 | 18.94883992 | 39 | 287 | 241 |
| 2 | OAR2_208652510 | 197056568 | 14.28969085 | 289 | 45 | 232 |
| 2 | OAR2_209720831 | 198155571 | 16.90009856 | 188 | 97 | 280 |
| 2 | OAR2_214771311 | 202942570 | 14.77241406 | 339 | 37 | 191 |
| 2 | OAR2_215421386 | 203665902 | 13.88386339 | 473 | 3 | 91 |
| 2 | OAR2_241633258 | 228848890 | 16.38584048 | 29 | 327 | 211 |
| 3 | s44996 | 143455 | 17.79117627 | 357 | 15 | 195 |
| 3 | s26048 | 199397 | 17.52845486 | 375 | 12 | 180 |
| 3 | s26968 | 42837449 | 17.79113051 | 421 | 6 | 140 |
| 3 | OAR3_66048281 | 62424338 | 14.62008359 | 70 | 230 | 267 |
| 3 | OAR3_70378885 | 66588993 | 16.23022316 | 122 | 160 | 284 |
| 3 | OAR3_70578986 | 66786673 | 14.17726891 | 218 | 87 | 262 |
| 3 | OAR3_71334336 | 67486045 | 14.17958765 | 84 | 219 | 264 |
| 3 | OAR3_73971642 | 69981369 | 14.23667517 | 380 | 20 | 166 |
| 3 | OAR3_75600301 | 71501518 | 14.38026083 | 134 | 140 | 293 |
| 3 | OAR3_79513396 | 75299649 | 14.22959647 | 121 | 165 | 281 |
| 3 | OAR3_88546737 | 83625995 | 14.79436674 | 254 | 54 | 255 |
| 3 | OAR3_90027100 | 85079269 | 18.0215431 | 380 | 21 | 166 |
| 3 | s51570 | 105949897 | 14.03532278 | 34 | 335 | 198 |
| 3 | OAR3_120348297 | 112892904 | 17.22481059 | 113 | 145 | 308 |
| 3 | OAR3_121655834 | 114073925 | 14.36327069 | 87 | 192 | 255 |
| 3 | OAR3_122117752 | 114556005 | 13.92969724 | 347 | 38 | 182 |
| 3 | s24987 | 115230555 | 14.77682707 | 40 | 300 | 227 |
| 3 | OAR3_125588829 | 117721356 | 17.62737815 | 78 | 197 | 284 |
| 3 | OAR3_127180087 | 119223799 | 14.78247827 | 314 | 45 | 204 |
| 3 | OAR3_130672189 | 122602823 | 14.07914172 | 3 | 460 | 71 |
| 3 | OAR3_149613458 | 139826948 | 13.55067865 | 112 | 171 | 284 |
| 3 | OAR3_158745008_X | 148888684 | 13.7834001 | 137 | 143 | 287 |
| 3 | s40475 | 149468411 | 14.72361773 | 28 | 338 | 201 |
| 3 | OAR3_159525447 | 149598572 | 18.32194811 | 75 | 234 | 254 |
| 3 | OAR3_159602580 | 149673526 | 15.07255828 | 55 | 287 | 223 |
| 3 | OAR3_159624684 | 149692512 | 21.1026353 | 113 | 178 | 276 |
| 3 | OAR3_166345320 | 155485554 | 14.17146637 | 76 | 220 | 268 |
| 3 | OAR3_167586056 | 156613118 | 18.96161076 | 407 | 17 | 142 |
| 3 | OAR3_172603806 | 161433276 | 14.4951318 | 164 | 127 | 276 |
| 3 | OAR3_172660325 | 161487035 | 15.80880003 | 225 | 74 | 268 |
| 3 | s29697 | 161598677 | 13.83756973 | 144 | 157 | 265 |
| 3 | OAR3_180858156 | 168402718 | 13.98820412 | 133 | 143 | 256 |
| 3 | OAR3_184736236 | 172136657 | 20.93566595 | 99 | 188 | 272 |
| 3 | OAR3_204157824 | 189580068 | 13.92892966 | 368 | 19 | 180 |
| 3 | s70082 | 190881196 | 18.00807034 | 46 | 296 | 220 |
| 3 | OAR3_205527658 | 190907829 | 14.41711136 | 248 | 71 | 248 |
| 3 | OAR3_206007678 | 191323349 | 15.41572942 | 32 | 300 | 201 |
| 3 | OAR3_208879910 | 194174324 | 15.07894697 | 484 | 5 | 77 |
| 3 | OAR3_212110574 | 196928388 | 16.85088459 | 248 | 55 | 264 |
| 3 | OAR3_212476314_X | 197308620 | 14.75725833 | 204 | 89 | 274 |
| 3 | OAR3_214809983 | 199630233 | 15.2725947 | 5 | 471 | 91 |
| 3 | OAR3_218072179 | 202543162 | 16.02825118 | 82 | 215 | 269 |
| 3 | OAR3_218425833 | 202913579 | 16.79298992 | 444 | 7 | 116 |
| 3 | s11874 | 216940930 | 13.91854823 | 31 | 344 | 191 |
| 4 | OAR4_15883493 | 15636014 | 13.58625416 | 168 | 117 | 282 |
| 4 | s75938 | 16448024 | 14.43923062 | 69 | 217 | 281 |
| 4 | OAR4_27853334 | 26449971 | 16.50941183 | 159 | 117 | 291 |
| 4 | OAR4_28162430 | 26767933 | 13.72036001 | 172 | 107 | 288 |
| 4 | OAR4_30718809 | 29221112 | 20.02016855 | 17 | 374 | 176 |
| 4 | OAR4_31079268 | 29568108 | 17.78599153 | 17 | 364 | 186 |
| 4 | OAR4_32059279 | 30498505 | 15.33373041 | 13 | 388 | 166 |
| 4 | OAR4_33401766_X | 31767262 | 13.93082488 | 76 | 224 | 267 |
| 4 | OAR4_37455919 | 35463534 | 16.48637938 | 304 | 42 | 217 |
| 4 | OAR4_38036161 | 36053171 | 14.11212005 | 48 | 273 | 246 |
| 4 | s65396 | 36358273 | 15.74082495 | 132 | 159 | 276 |
| 4 | OAR4_38383717 | 36395312 | 15.81437738 | 325 | 30 | 207 |
| 4 | OAR4_39494974 | 37402484 | 14.72691038 | 165 | 118 | 284 |
| 4 | OAR4_39505430 | 37414852 | 15.23789738 | 196 | 98 | 273 |
| 4 | OAR4_41703426 | 39577763 | 16.13175285 | 299 | 34 | 234 |
| 4 | OAR4_43261444 | 41160751 | 19.26306046 | 47 | 258 | 251 |
| 4 | OAR4_46498577 | 44060327 | 17.03551243 | 188 | 92 | 287 |
| 4 | OAR4_46599830 | 44153679 | 16.76153906 | 21 | 359 | 187 |
| 4 | OAR4_47315855 | 44762301 | 17.00263291 | 17 | 366 | 184 |
| 4 | OAR4_47332566 | 44779133 | 14.97013152 | 387 | 13 | 165 |
| 4 | s06433 | 46673082 | 15.35259523 | 409 | 5 | 150 |
| 4 | s43001 | 47089935 | 13.99212515 | 432 | 5 | 130 |
| 4 | OAR4_51690410_X | 48864585 | 18.83042763 | 151 | 132 | 283 |
| 4 | OAR4_53108425 | 50129083 | 20.12163828 | 267 | 61 | 239 |
| 4 | OAR4_53229514 | 50302948 | 14.46740308 | 23 | 352 | 192 |
| 4 | OAR4_53437353 | 50496365 | 13.88724097 | 29 | 334 | 204 |
| 4 | OAR4_54817254_X | 51724251 | 13.8846993 | 12 | 427 | 128 |
| 4 | OAR4_56284966 | 53150948 | 15.23211948 | 20 | 369 | 178 |
| 4 | OAR4_56364872 | 53235300 | 16.71411857 | 213 | 73 | 281 |
| 4 | OAR4_60302322 | 57102148 | 14.30142234 | 153 | 113 | 267 |
| 4 | OAR4_62748691 | 59406742 | 16.68127207 | 90 | 180 | 286 |
| 4 | OAR4_62820882 | 59470198 | 13.69848356 | 135 | 149 | 253 |
| 4 | OAR4_64413453 | 60892228 | 14.45919101 | 220 | 72 | 274 |
| 4 | OAR4_67814662 | 64072450 | 17.13753626 | 196 | 98 | 217 |
| 4 | s49944 | 64135978 | 18.18882751 | 92 | 206 | 268 |
| 4 | s46443 | 65413387 | 13.67488752 | 453 | 7 | 107 |
| 4 | s22015 | 76449937 | 13.54904962 | 363 | 18 | 153 |
| 4 | OAR4_92963794 | 87542688 | 13.8505782 | 132 | 143 | 289 |
| 4 | OAR4_96077733 | 90539210 | 13.52890604 | 77 | 224 | 266 |
| 4 | s12931 | 95835685 | 15.12811743 | 194 | 91 | 273 |
| 4 | s04041 | 98642141 | 16.44685601 | 184 | 95 | 288 |
| 4 | OAR4_105692408 | 99304591 | 14.64273215 | 193 | 94 | 247 |
| 4 | OAR4_107741257 | 101268547 | 19.52754663 | 188 | 102 | 277 |
| 4 | OAR4_118091493 | 110621610 | 15.54940688 | 6 | 448 | 113 |
| 5 | s16712 | 827819 | 13.85434999 | 11 | 409 | 146 |
| 5 | OAR5_6477945 | 6200590 | 13.92791203 | 4 | 447 | 115 |
| 5 | s71821 | 39307479 | 13.91592436 | 381 | 16 | 170 |
| 5 | s15115 | 64627238 | 14.39797829 | 3 | 456 | 105 |
| 5 | s29854 | 79322514 | 13.78850327 | 181 | 97 | 289 |
| 5 | OAR5_88047810 | 80107598 | 15.24236553 | 166 | 119 | 278 |
| 5 | OAR5_94157237 | 85947285 | 13.62628994 | 387 | 23 | 157 |
| 5 | OAR5_94671857 | 86576008 | 13.86356968 | 426 | 6 | 135 |
| 5 | OAR5_102931003 | 94471264 | 14.32323283 | 170 | 88 | 273 |
| 5 | OAR5_104164895 | 95711861 | 13.98345855 | 118 | 167 | 282 |
| 5 | OAR5_105151936 | 96576753 | 14.37057007 | 40 | 304 | 222 |
| 5 | OAR5_112227256 | 103087994 | 13.66265804 | 7 | 468 | 92 |
| 5 | OAR5_112871826 | 103715540 | 16.9462297 | 221 | 71 | 269 |
| 6 | OAR6_6278702 | 4253258 | 14.8455859 | 56 | 250 | 260 |
| 6 | OAR6_12582717 | 10086493 | 15.97678262 | 364 | 22 | 181 |
| 6 | s62995 | 13178916 | 16.51543787 | 164 | 114 | 287 |
| 6 | OAR6_19956589 | 17038514 | 13.56490621 | 195 | 88 | 284 |
| 6 | OAR6_22542410 | 19613700 | 13.72996001 | 29 | 326 | 212 |
| 6 | OAR6_22649353 | 19706998 | 13.88348101 | 389 | 16 | 162 |
| 6 | OAR6_31922774 | 28123410 | 17.51476989 | 146 | 146 | 275 |
| 6 | OAR6_32516276 | 28676510 | 26.85610976 | 94 | 181 | 292 |
| 6 | OAR6_32535566 | 28698320 | 16.81596968 | 24 | 369 | 174 |
| 6 | OAR6_32661836 | 28829203 | 18.35326814 | 40 | 266 | 261 |
| 6 | s71628 | 29009593 | 18.41454097 | 92 | 217 | 258 |
| 6 | OAR6_33291928 | 29450053 | 18.49925716 | 443 | 10 | 114 |
| 6 | OAR6_34895948 | 30936764 | 17.125545 | 161 | 152 | 253 |
| 6 | OAR6_35293995 | 31247619 | 16.56520627 | 138 | 135 | 294 |
| 6 | OAR6_35458562 | 31416704 | 16.83815553 | 108 | 164 | 292 |
| 6 | OAR6_35622056 | 31590979 | 15.47673247 | 340 | 25 | 200 |
| 6 | OAR6_37176737 | 33197652 | 14.4496928 | 104 | 188 | 275 |
| 6 | OAR6_37459560 | 33467474 | 15.19790521 | 333 | 28 | 204 |
| 6 | OAR6_37750101 | 33770695 | 16.81659089 | 210 | 63 | 253 |
| 6 | OAR6_38315830 | 34256151 | 24.58107617 | 321 | 24 | 222 |
| 6 | OAR6_38496068 | 34442216 | 13.53751626 | 19 | 349 | 199 |
| 6 | OAR6_38840563 | 34712230 | 20.22980676 | 266 | 64 | 234 |
| 6 | OAR6_38863569 | 34744791 | 18.19788777 | 38 | 308 | 221 |
| 6 | OAR6_38919831 | 34800345 | 16.22527315 | 307 | 38 | 221 |
| 6 | s43499 | 36008443 | 13.83618307 | 13 | 405 | 149 |
| 6 | OAR6_40243862 | 36040916 | 14.77753962 | 21 | 385 | 161 |
| 6 | OAR6_40277406 | 36073405 | 31.76834021 | 89 | 202 | 275 |
| 6 | s73850 | 36461468 | 14.47623498 | 358 | 13 | 196 |
| 6 | OAR6_40855809 | 36655091 | 33.11562675 | 76 | 229 | 262 |
| 6 | OAR6_40955920 | 36750972 | 22.68512016 | 72 | 238 | 257 |
| 6 | DU178311_404 | 36839547 | 28.22751773 | 244 | 56 | 267 |
| 6 | s32980 | 36914376 | 15.51399552 | 371 | 25 | 171 |
| 6 | OAR6_41192387 | 37015361 | 15.83228987 | 324 | 30 | 211 |
| 6 | s17946 | 37164263 | 16.74218333 | 26 | 341 | 200 |
| 6 | OAR6_41424992 | 37203894 | 17.29911447 | 17 | 362 | 186 |
| 6 | OAR6_41558126 | 37334387 | 18.51037357 | 301 | 47 | 218 |
| 6 | OAR6_41768532 | 37533664 | 16.07399367 | 397 | 13 | 157 |
| 6 | OAR6_41850329 | 37615241 | 15.36206943 | 339 | 25 | 201 |
| 6 | OAR6_41877997 | 37640732 | 15.76864532 | 416 | 11 | 140 |
| 6 | OAR6_42094768 | 37832085 | 15.88965408 | 8 | 412 | 147 |
| 6 | OAR6_42447403 | 38180902 | 17.29157679 | 318 | 20 | 229 |
| 6 | OAR6_42576838 | 38310652 | 21.69052297 | 78 | 220 | 269 |
| 6 | OAR6_43292772 | 38906528 | 14.8125746 | 156 | 138 | 273 |
| 6 | OAR6_45040786 | 40356705 | 16.59099605 | 9 | 439 | 119 |
| 6 | OAR6_45285663 | 40594243 | 17.23786872 | 132 | 146 | 289 |
| 6 | OAR6_45574191 | 40887941 | 15.60737146 | 67 | 247 | 252 |
| 6 | OAR6_45662944 | 40975597 | 27.29849671 | 377 | 10 | 180 |
| 6 | OAR6_45740327 | 41049741 | 19.86528852 | 65 | 249 | 232 |
| 6 | OAR6_46243783 | 41507029 | 18.19544292 | 315 | 25 | 194 |
| 6 | OAR6_46292636 | 41543522 | 15.33236812 | 183 | 94 | 290 |
| 6 | s72414 | 43753893 | 14.95849402 | 45 | 298 | 224 |
| 6 | s08703 | 44306250 | 14.07467865 | 127 | 154 | 272 |
| 6 | OAR6_49871298 | 44939727 | 17.75225957 | 325 | 32 | 207 |
| 6 | OAR6_49878575 | 44950513 | 18.31483685 | 33 | 327 | 207 |
| 6 | OAR6_61499652 | 55785560 | 15.40724304 | 235 | 57 | 275 |
| 6 | OAR6_64452456 | 58478983 | 13.90683113 | 181 | 117 | 268 |
| 6 | OAR6_65010015 | 59028252 | 16.81035754 | 31 | 334 | 169 |
| 6 | s47899 | 59467273 | 13.6728703 | 62 | 225 | 279 |
| 6 | s13347 | 60727514 | 17.76627264 | 10 | 421 | 136 |
| 6 | OAR6_68249537 | 62129559 | 15.09507884 | 284 | 33 | 250 |
| 6 | OAR6_68773720 | 62634644 | 16.74834128 | 30 | 310 | 226 |
| 6 | OAR6_69567467 | 63101221 | 13.92902031 | 399 | 20 | 148 |
| 6 | OAR6_69574450 | 63107762 | 14.91562715 | 19 | 399 | 148 |
| 6 | OAR6_70425725 | 64144346 | 16.9206261 | 262 | 43 | 254 |
| 6 | OAR6_72885011 | 66463797 | 13.69962161 | 3 | 452 | 112 |
| 6 | OAR6_76284920 | 69798999 | 16.17045476 | 307 | 35 | 223 |
| 6 | OAR6_76793301 | 70359799 | 13.66848583 | 30 | 340 | 185 |
| 6 | OAR6_76858399 | 70430615 | 16.53409142 | 89 | 189 | 275 |
| 6 | OAR6_79378917 | 72795375 | 13.68884283 | 75 | 228 | 264 |
| 6 | OAR6_81094573 | 74275591 | 15.24699126 | 27 | 357 | 183 |
| 6 | OAR6_85127413 | 77973305 | 15.23949136 | 86 | 200 | 281 |
| 6 | OAR6_85367529 | 78194522 | 16.87100228 | 286 | 49 | 231 |
| 6 | OAR6_85380309 | 78209559 | 18.326664 | 64 | 258 | 245 |
| 6 | OAR6_86918946 | 79601311 | 14.32741541 | 378 | 21 | 168 |
| 6 | OAR6_88512031 | 81071327 | 14.30973165 | 106 | 174 | 287 |
| 6 | OAR6_93266572 | 85018309 | 15.57655108 | 415 | 9 | 143 |
| 6 | OAR6_95454480 | 87098156 | 14.24055126 | 35 | 293 | 239 |
| 6 | OAR6_95758613 | 87410801 | 14.22351523 | 414 | 11 | 142 |
| 6 | OAR6_95813186 | 87465916 | 15.08746033 | 327 | 23 | 216 |
| 6 | OAR6_102416653 | 93407497 | 14.51254162 | 218 | 85 | 264 |
| 6 | OAR6_103110795 | 94100798 | 15.55491992 | 140 | 130 | 297 |
| 6 | s03639 | 94198327 | 14.43056361 | 12 | 425 | 130 |
| 6 | OAR6_104194705 | 94934431 | 13.94000446 | 8 | 447 | 112 |
| 6 | OAR6_120127831 | 105674424 | 13.70081765 | 396 | 14 | 156 |
| 6 | OAR6_120632773 | 106190183 | 15.9334669 | 15 | 392 | 160 |
| 6 | OAR6_123771724 | 108872442 | 13.9002057 | 84 | 206 | 277 |
| 7 | OAR7_8371742 | 8410720 | 16.63838496 | 88 | 193 | 286 |
| 7 | OAR7_8381203 | 8420101 | 13.83682896 | 254 | 51 | 262 |
| 7 | OAR7_9303645 | 9290672 | 13.52177767 | 109 | 180 | 275 |
| 7 | s02711 | 17193574 | 14.33030543 | 138 | 128 | 301 |
| 7 | s52058 | 17834128 | 13.72121931 | 337 | 28 | 201 |
| 7 | OAR7_18853420 | 18122564 | 19.5947479 | 77 | 215 | 275 |
| 7 | OAR7_18874301 | 18143257 | 17.11605884 | 69 | 226 | 269 |
| 7 | OAR7_19803272 | 18990754 | 14.44318334 | 74 | 259 | 234 |
| 7 | OAR7_19813387 | 19002938 | 17.27512462 | 303 | 45 | 219 |
| 7 | OAR7_19928730 | 19115796 | 14.95503513 | 245 | 70 | 252 |
| 7 | s58125 | 20153292 | 18.65140063 | 292 | 55 | 220 |
| 7 | s18314 | 20292100 | 13.66649982 | 25 | 327 | 215 |
| 7 | OAR7_21639936 | 20784154 | 14.01644819 | 278 | 45 | 239 |
| 7 | s22326 | 21169136 | 17.33542694 | 78 | 220 | 268 |
| 7 | OAR7_22744617 | 21820321 | 13.97642081 | 254 | 57 | 255 |
| 7 | s59897 | 33450952 | 14.52194445 | 416 | 12 | 139 |
| 7 | OAR7_49880260 | 45145205 | 15.09835982 | 21 | 399 | 147 |
| 7 | OAR7_52259603 | 47385334 | 16.69626258 | 29 | 332 | 206 |
| 7 | OAR7_52266557 | 47391463 | 20.08091154 | 117 | 152 | 296 |
| 7 | OAR7_53056448 | 48000419 | 13.91748358 | 171 | 116 | 277 |
| 7 | OAR7_53145734 | 48085586 | 19.97933759 | 167 | 116 | 282 |
| 7 | OAR7_53659419_X | 48597074 | 15.34295469 | 92 | 207 | 268 |
| 7 | OAR7_53952286 | 48854441 | 14.86091426 | 175 | 102 | 290 |
| 7 | OAR7_53985984 | 48887292 | 15.81619056 | 101 | 176 | 290 |
| 7 | DU398082_567 | 48962917 | 17.11378081 | 70 | 199 | 287 |
| 7 | OAR7_59478261 | 53832042 | 13.72542777 | 21 | 355 | 191 |
| 7 | OAR7_68978948 | 63115852 | 13.62656185 | 196 | 114 | 257 |
| 7 | OAR7_69482785 | 63522691 | 13.98321114 | 75 | 237 | 255 |
| 7 | OAR7_70071978 | 64009832 | 14.3984338 | 255 | 59 | 253 |
| 7 | s01854 | 67238720 | 16.69573009 | 192 | 82 | 293 |
| 7 | OAR7_74095346 | 67556667 | 14.34018969 | 84 | 181 | 297 |
| 7 | OAR7_78744948 | 71889975 | 14.54443065 | 263 | 54 | 248 |
| 7 | OAR7_79067545 | 72210142 | 15.98656749 | 299 | 32 | 232 |
| 7 | OAR7_86742692 | 79502628 | 16.99134861 | 316 | 39 | 211 |
| 7 | OAR7_86706855 | 79534450 | 16.68423837 | 310 | 39 | 208 |
| 7 | OAR7_87417950 | 80047845 | 14.44703744 | 166 | 113 | 288 |
| 7 | s47068 | 80185886 | 13.52066897 | 152 | 132 | 281 |
| 7 | OAR7_87837965 | 80421010 | 14.96693227 | 193 | 92 | 282 |
| 7 | OAR7_90890170 | 83510096 | 16.75468764 | 161 | 115 | 291 |
| 7 | s17871 | 84699340 | 16.04562227 | 113 | 164 | 272 |
| 7 | s34501 | 84728573 | 17.92629776 | 379 | 19 | 168 |
| 7 | s60702 | 84994701 | 14.24564546 | 42 | 308 | 217 |
| 7 | s23968 | 85677888 | 14.56348309 | 207 | 100 | 260 |
| 7 | s02190 | 85693486 | 17.13504116 | 24 | 387 | 156 |
| 7 | s43612 | 86188592 | 13.85898052 | 186 | 112 | 269 |
| 7 | s69910 | 86451406 | 14.32354256 | 387 | 9 | 166 |
| 7 | s04294 | 86568727 | 15.02043859 | 420 | 11 | 134 |
| 7 | s62770 | 87119393 | 14.0206811 | 278 | 48 | 241 |
| 7 | OAR7_96641220 | 88828364 | 15.53207591 | 311 | 35 | 221 |
| 7 | OAR7_96756883 | 88936178 | 17.19747307 | 5 | 423 | 106 |
| 7 | OAR7_97127242 | 89263176 | 15.78711066 | 31 | 313 | 217 |
| 7 | OAR7_100406183 | 92215284 | 14.26896057 | 428 | 8 | 131 |
| 7 | OAR7_103688806 | 95175675 | 13.76561167 | 148 | 127 | 289 |
| 8 | OAR8_2389184 | 2165227 | 15.04080546 | 466 | 4 | 97 |
| 8 | OAR8_3752643 | 3446101 | 17.04768976 | 129 | 131 | 306 |
| 8 | OAR8_10787192 | 9644914 | 14.24225558 | 73 | 229 | 265 |
| 8 | s22824 | 29887855 | 15.11918829 | 321 | 27 | 219 |
| 8 | OAR8_32384391 | 29947895 | 14.91354927 | 304 | 34 | 229 |
| 8 | OAR8_36781700 | 34177033 | 15.15205637 | 18 | 373 | 176 |
| 8 | OAR8_40819881 | 37875271 | 13.64474198 | 14 | 390 | 163 |
| 8 | OAR8_42165866 | 39201965 | 16.53595347 | 161 | 128 | 278 |
| 8 | OAR8_44326031_X | 41224885 | 14.9514463 | 450 | 7 | 110 |
| 8 | OAR8_50006699 | 46540498 | 15.17865269 | 320 | 42 | 205 |
| 8 | s05996 | 47298216 | 14.73360027 | 32 | 314 | 221 |
| 8 | OAR8_53119810 | 49560934 | 14.03231336 | 345 | 25 | 191 |
| 8 | OAR8_54418806 | 50783340 | 14.93930265 | 380 | 27 | 160 |
| 8 | OAR8_56107289 | 52190725 | 13.50674734 | 67 | 239 | 261 |
| 8 | OAR8_58959092 | 55029049 | 16.34839869 | 9 | 384 | 174 |
| 8 | s42849 | 56844164 | 13.73301034 | 250 | 47 | 261 |
| 8 | s21279 | 73328996 | 18.1250371 | 23 | 340 | 204 |
| 8 | s63436 | 88325082 | 14.09881644 | 72 | 209 | 286 |
| 9 | OAR9_6158862 | 6280780 | 13.95931234 | 337 | 31 | 199 |
| 9 | OAR9_9370813_X | 9499917 | 14.88368124 | 322 | 29 | 213 |
| 9 | s37809 | 14485051 | 13.96429346 | 267 | 47 | 253 |
| 9 | s22485 | 15930086 | 17.23065033 | 233 | 66 | 259 |
| 9 | s35717 | 16554266 | 13.77562201 | 44 | 271 | 250 |
| 9 | OAR9_25222765 | 24253155 | 13.96058185 | 15 | 382 | 169 |
| 9 | OAR9_34532217 | 32926251 | 14.27017703 | 217 | 86 | 264 |
| 9 | OAR9_41768159 | 39730763 | 17.31545617 | 393 | 13 | 155 |
| 9 | s14503 | 55984413 | 13.85858929 | 376 | 15 | 175 |
| 9 | OAR9_71225808 | 67204995 | 13.71222499 | 48 | 288 | 231 |
| 9 | OAR9_92302903 | 87093273 | 13.60336779 | 29 | 326 | 212 |
| 9 | OAR9_94815557 | 89207981 | 14.78235004 | 94 | 199 | 274 |
| 9 | OAR9_94983893 | 89390357 | 14.59491848 | 367 | 21 | 176 |
| 9 | OAR9_95152473 | 89573491 | 14.03324282 | 3 | 461 | 103 |
| 9 | OAR9_95284529 | 89706814 | 18.78046393 | 357 | 23 | 185 |
| 9 | DU471913_499 | 90950993 | 16.36689734 | 208 | 89 | 261 |
| 10 | OAR10_8668332_X | 10304928 | 17.92485181 | 64 | 240 | 262 |
| 10 | s62366 | 11974059 | 13.792091 | 10 | 407 | 147 |
| 10 | OAR10_26266695 | 26350351 | 14.51434453 | 80 | 234 | 252 |
| 10 | OAR10_27832232 | 27809739 | 17.82849443 | 418 | 7 | 142 |
| 10 | OAR10_27878618 | 27854262 | 17.82849443 | 7 | 418 | 142 |
| 10 | OAR10_49717223 | 48923202 | 18.51551571 | 72 | 181 | 279 |
| 10 | s41516 | 53341344 | 14.21970274 | 85 | 191 | 291 |
| 10 | OAR10_60388029 | 58966248 | 13.76350399 | 3 | 440 | 124 |
| 10 | OAR10_76484955_X | 71470411 | 13.77813481 | 261 | 58 | 248 |
| 10 | OAR10_79482502 | 72600246 | 16.18049659 | 40 | 300 | 227 |
| 10 | OAR10_80164964 | 73238801 | 13.91982078 | 98 | 187 | 280 |
| 10 | s53758 | 73484288 | 17.36177444 | 121 | 155 | 290 |
| 10 | s60785 | 73945414 | 13.75120523 | 233 | 58 | 276 |
| 10 | s01281 | 74111393 | 15.52021946 | 201 | 84 | 282 |
| 10 | s17895 | 74969498 | 13.52337548 | 115 | 152 | 296 |
| 10 | s12406 | 86343287 | 14.24669493 | 346 | 24 | 189 |
| 10 | OAR10_94127923 | 86415081 | 18.07633843 | 345 | 26 | 195 |
| 11 | s48201 | 17165413 | 15.73531436 | 6 | 446 | 114 |
| 11 | s47275 | 19490505 | 13.68586456 | 465 | 5 | 97 |
| 11 | OAR11_22045347 | 21292420 | 16.30993155 | 464 | 7 | 96 |
| 11 | s08572 | 21848495 | 15.17262525 | 6 | 435 | 126 |
| 11 | s21891 | 23997615 | 13.65925176 | 277 | 47 | 236 |
| 11 | OAR11_32227171 | 30350640 | 16.11880389 | 73 | 217 | 277 |
| 11 | s23269 | 32379091 | 15.1817052 | 20 | 366 | 180 |
| 11 | OAR11_35075289 | 32840694 | 14.20937518 | 74 | 221 | 271 |
| 11 | OAR11_35176999 | 32942352 | 13.85656676 | 222 | 74 | 271 |
| 11 | OAR11_35522612 | 33267847 | 16.18301711 | 343 | 30 | 194 |
| 11 | OAR11_37481624 | 35133720 | 22.90759344 | 22 | 399 | 146 |
| 11 | OAR11_37550019 | 35199669 | 21.24466166 | 62 | 263 | 242 |
| 11 | s06509 | 36582847 | 20.6147503 | 234 | 80 | 251 |
| 11 | s50867 | 37075696 | 15.1402486 | 98 | 195 | 271 |
| 11 | s48741 | 37147168 | 23.26894523 | 337 | 28 | 202 |
| 11 | s10472 | 37170224 | 16.64552296 | 76 | 248 | 243 |
| 11 | s09803 | 38319263 | 14.99784679 | 151 | 140 | 269 |
| 11 | OAR11_41358436 | 38914719 | 14.66169274 | 197 | 87 | 281 |
| 11 | OAR11_41384177 | 38939895 | 17.08744487 | 443 | 4 | 117 |
| 11 | OAR11_41967031 | 39492256 | 13.95471338 | 16 | 359 | 192 |
| 11 | s40291 | 41530998 | 16.47489308 | 57 | 262 | 248 |
| 11 | OAR11_49921941 | 46928316 | 13.58225005 | 150 | 140 | 277 |
| 11 | OAR11_50969324 | 47979791 | 14.44351988 | 480 | 5 | 82 |
| 11 | OAR11_53671543 | 50313519 | 14.02527883 | 45 | 284 | 238 |
| 11 | s30915 | 51479563 | 15.97362789 | 438 | 8 | 121 |
| 12 | OAR12_16086297 | 13419002 | 14.17319662 | 444 | 7 | 116 |
| 12 | OAR12_31477278 | 27961834 | 13.616672 | 3 | 498 | 64 |
| 12 | OAR12_33050823 | 29449529 | 17.53345196 | 420 | 11 | 136 |
| 12 | s16442 | 45881414 | 15.92258683 | 117 | 185 | 254 |
| 12 | OAR12_51109106 | 46187849 | 14.50193791 | 423 | 7 | 137 |
| 12 | OAR12_56663780 | 51233503 | 14.84730337 | 191 | 92 | 281 |
| 12 | OAR12_62551076 | 56204038 | 15.77944429 | 157 | 112 | 292 |
| 12 | OAR12_64628319 | 58250035 | 15.60811904 | 3 | 479 | 85 |
| 12 | OAR12_64996265 | 58613284 | 18.7777931 | 380 | 13 | 173 |
| 12 | s41819 | 60695474 | 17.23913708 | 331 | 25 | 208 |
| 12 | OAR12_70479451 | 63960016 | 13.53554432 | 35 | 314 | 218 |
| 12 | s60193 | 67057048 | 14.18063696 | 64 | 249 | 234 |
| 12 | OAR12_78952832 | 71644661 | 17.47951255 | 19 | 356 | 159 |
| 12 | s72421 | 78133465 | 18.31472229 | 274 | 44 | 248 |
| 13 | OAR13_5988822 | 5208762 | 13.7582528 | 264 | 38 | 254 |
| 13 | OAR13_33896421 | 30733899 | 14.08975787 | 230 | 66 | 262 |
| 13 | OAR13_34394544 | 31219163 | 13.62690509 | 102 | 166 | 298 |
| 13 | s42220 | 44193791 | 16.6391331 | 127 | 156 | 282 |
| 13 | s69808 | 46333109 | 19.50053534 | 299 | 36 | 232 |
| 13 | OAR13_51727898 | 48493120 | 15.2654056 | 7 | 448 | 112 |
| 13 | OAR13_57705920 | 53008906 | 15.31387362 | 46 | 258 | 235 |
| 13 | OAR13_58685132 | 53934510 | 15.01759658 | 310 | 28 | 221 |
| 13 | OAR13_58977999 | 54219758 | 15.2208357 | 212 | 70 | 279 |
| 13 | s46535 | 54711166 | 17.79373175 | 337 | 36 | 193 |
| 13 | OAR13_61475864 | 56502268 | 15.31202173 | 16 | 408 | 142 |
| 13 | s68335 | 56853591 | 13.86413998 | 214 | 86 | 267 |
| 13 | OAR13_62738272 | 57604481 | 15.52084384 | 16 | 381 | 165 |
| 13 | s56953 | 60375605 | 16.60340983 | 24 | 333 | 210 |
| 13 | s11373 | 61563593 | 13.99873913 | 142 | 149 | 276 |
| 13 | s51670 | 62767123 | 15.29951141 | 410 | 11 | 146 |
| 13 | OAR13_69559303_X | 64482148 | 14.00653768 | 204 | 73 | 290 |
| 13 | s67702 | 66132902 | 17.22960247 | 17 | 359 | 191 |
| 14 | s31805 | 1120187 | 14.69947194 | 14 | 387 | 166 |
| 14 | s53546 | 2899898 | 14.78051241 | 40 | 267 | 258 |
| 14 | OAR14_4021588 | 4081215 | 14.32054445 | 172 | 103 | 292 |
| 14 | OAR14_4313360 | 4382354 | 19.46922453 | 138 | 159 | 269 |
| 14 | s50332 | 12886296 | 13.62903711 | 124 | 146 | 296 |
| 14 | s02394 | 19997991 | 14.40577919 | 263 | 46 | 258 |
| 14 | OAR14_31424342 | 30192766 | 17.48341121 | 197 | 80 | 290 |
| 14 | OAR14_32139675 | 30908494 | 15.50174796 | 60 | 255 | 232 |
| 14 | s50600 | 37861999 | 18.04461232 | 141 | 150 | 262 |
| 14 | OAR14_39624987 | 38043283 | 16.81386528 | 174 | 100 | 292 |
| 14 | OAR14_41476687 | 39795104 | 15.61765738 | 336 | 27 | 202 |
| 14 | OAR14_41540680_X | 39858010 | 13.78114774 | 336 | 23 | 201 |
| 14 | s42198 | 39988306 | 17.28953807 | 95 | 200 | 272 |
| 14 | DU513272_396 | 43852799 | 19.03630832 | 24 | 325 | 218 |
| 14 | s14023 | 44744891 | 13.60210832 | 421 | 4 | 142 |
| 14 | OAR14_48298313 | 45985867 | 15.4601884 | 113 | 173 | 281 |
| 14 | OAR14_49049707 | 46701106 | 16.16497816 | 128 | 117 | 286 |
| 14 | OAR14_53406640 | 50504964 | 14.56169515 | 325 | 28 | 212 |
| 14 | OAR14_54961126 | 52010672 | 13.9845015 | 228 | 62 | 269 |
| 14 | OAR14_62158996 | 57583997 | 13.51564227 | 13 | 388 | 161 |
| 15 | OAR15_2886961 | 3462742 | 14.64596131 | 21 | 369 | 176 |
| 15 | OAR15_19808569 | 19110832 | 14.29969337 | 72 | 194 | 268 |
| 15 | s52387 | 19429799 | 20.15498579 | 17 | 393 | 157 |
| 15 | OAR15_26197833 | 25047590 | 14.44338277 | 74 | 241 | 252 |
| 15 | s33841 | 25702962 | 17.05574304 | 330 | 21 | 216 |
| 15 | s47819 | 25848489 | 14.61799135 | 7 | 440 | 120 |
| 15 | OAR15_39626442 | 37776247 | 13.58734476 | 165 | 111 | 284 |
| 15 | s40251 | 37925945 | 16.53910311 | 35 | 334 | 198 |
| 15 | OAR15_39821855_X | 37967824 | 15.87989151 | 205 | 82 | 280 |
| 15 | OAR15_45082881 | 42930293 | 14.48978622 | 188 | 106 | 273 |
| 15 | OAR15_46408810 | 44159810 | 13.9203386 | 429 | 12 | 126 |
| 15 | s74416 | 46354475 | 14.77930712 | 391 | 15 | 161 |
| 15 | s00681 | 52030766 | 14.68467893 | 127 | 143 | 296 |
| 15 | s43110 | 52741951 | 13.71387311 | 5 | 465 | 97 |
| 15 | s31847 | 52838617 | 14.35218391 | 425 | 11 | 131 |
| 15 | OAR15_61851386 | 56485935 | 13.61718784 | 20 | 336 | 207 |
| 15 | s36214 | 64690808 | 14.01836485 | 33 | 300 | 234 |
| 16 | OAR16_8135295 | 7635517 | 13.54270403 | 75 | 219 | 273 |
| 16 | s10381 | 7865471 | 15.29849814 | 31 | 345 | 191 |
| 16 | OAR16_28374637 | 26150633 | 18.00724942 | 81 | 205 | 270 |
| 16 | OAR16_34474012 | 31745899 | 14.74141912 | 3 | 458 | 106 |
| 16 | OAR16_36378409 | 33470561 | 14.10561748 | 106 | 180 | 279 |
| 16 | OAR16_50183420 | 45961427 | 16.00942217 | 21 | 333 | 213 |
| 16 | OAR16_55005404 | 50497537 | 13.9441351 | 297 | 39 | 231 |
| 16 | OAR16_55230534 | 50725124 | 18.94817474 | 301 | 34 | 229 |
| 16 | OAR16_55255370 | 50746672 | 17.15555024 | 304 | 32 | 231 |
| 16 | OAR16_56458897 | 51898799 | 13.7663761 | 37 | 307 | 223 |
| 16 | OAR16_56487742_X | 51930925 | 13.57267473 | 24 | 344 | 199 |
| 16 | OAR16_58262389 | 53439521 | 14.9824716 | 416 | 9 | 133 |
| 16 | OAR16_59197045 | 54287765 | 15.12547568 | 316 | 36 | 182 |
| 16 | OAR16_59809959 | 54922582 | 14.13634293 | 54 | 260 | 253 |
| 16 | s33640 | 61783963 | 14.06318735 | 183 | 107 | 272 |
| 16 | s53747 | 63548346 | 17.05264084 | 279 | 38 | 250 |
| 16 | s58116 | 63628552 | 15.12940529 | 146 | 138 | 250 |
| 16 | OAR16_72216389 | 66404779 | 18.58651795 | 75 | 204 | 288 |
| 16 | OAR16_73610347 | 67747341 | 17.75319095 | 45 | 317 | 204 |
| 16 | OAR16_73663651 | 67798858 | 14.53931898 | 379 | 8 | 138 |
| 16 | s57671 | 68563913 | 14.02410721 | 163 | 109 | 290 |
| 16 | s72149 | 68705421 | 19.05010324 | 53 | 273 | 239 |
| 16 | s70856 | 70101635 | 15.10713528 | 416 | 7 | 143 |
| 17 | s51152 | 51395628 | 13.74944599 | 122 | 167 | 275 |
| 17 | s54014 | 63024274 | 16.43685383 | 333 | 21 | 210 |
| 18 | OAR18_50905798 | 47704616 | 13.87961776 | 356 | 27 | 184 |
| 18 | OAR18_54327380 | 50830111 | 13.7664307 | 207 | 102 | 258 |
| 18 | s11355 | 63400549 | 14.16288952 | 39 | 306 | 220 |
| 19 | OAR19_4601190 | 4362142 | 13.6744771 | 144 | 131 | 292 |
| 19 | OAR19_4653492 | 4415925 | 17.55096312 | 75 | 215 | 277 |
| 19 | s34050 | 5216006 | 16.53129362 | 390 | 12 | 132 |
| 19 | s60865 | 7330076 | 16.39529691 | 313 | 48 | 206 |
| 19 | s50778 | 7642811 | 14.23756529 | 272 | 58 | 237 |
| 19 | OAR19_8122263_X | 7837780 | 14.41601023 | 179 | 121 | 266 |
| 19 | OAR19_8240039 | 7924739 | 17.19395835 | 231 | 81 | 255 |
| 19 | OAR19_10075837 | 9779153 | 21.66015231 | 187 | 117 | 263 |
| 19 | OAR19_10268706 | 9974257 | 13.72711146 | 158 | 108 | 300 |
| 19 | OAR19_12320210 | 11901045 | 21.07387189 | 31 | 322 | 213 |
| 19 | s45055 | 57014692 | 15.07767224 | 257 | 44 | 266 |
| 20 | OAR20_3067949 | 3118750 | 15.16051647 | 41 | 327 | 199 |
| 20 | OAR20_3201268 | 3260706 | 15.58715263 | 16 | 388 | 163 |
| 20 | OAR20_3937387 | 3943548 | 15.0883836 | 10 | 387 | 170 |
| 20 | s15165 | 8235497 | 13.51148408 | 397 | 9 | 161 |
| 20 | s54188 | 10282827 | 13.7520822 | 160 | 128 | 278 |
| 20 | s37693 | 11049634 | 17.11478342 | 373 | 14 | 180 |
| 20 | OAR20_12692128 | 12088750 | 13.88103341 | 58 | 258 | 251 |
| 20 | OAR20_12774774_X | 12133313 | 13.96754799 | 77 | 224 | 266 |
| 20 | OAR20_13938561 | 13220792 | 13.60611924 | 267 | 55 | 245 |
| 20 | OAR20_39342052 | 35900831 | 14.35527735 | 151 | 130 | 285 |
| 20 | s45918 | 48305665 | 14.18582545 | 111 | 155 | 301 |
| 21 | OAR21_7407668 | 6167907 | 13.79266279 | 358 | 15 | 160 |
| 21 | OAR21_8817818 | 7479728 | 14.40849476 | 153 | 114 | 298 |
| 21 | OAR21_15965129 | 13934267 | 20.69354845 | 425 | 13 | 127 |
| 21 | OAR21_16900072 | 14850333 | 16.85467193 | 15 | 372 | 176 |
| 21 | OAR21_24820440 | 22213120 | 14.26050646 | 96 | 212 | 257 |
| 21 | OAR21_25083552 | 22485141 | 14.13838877 | 439 | 5 | 119 |
| 21 | s69791 | 22973254 | 15.01313185 | 115 | 170 | 282 |
| 21 | OAR21_25685686 | 22982943 | 13.91230636 | 115 | 157 | 282 |
| 21 | OAR21_26091012 | 23321769 | 13.61118818 | 305 | 33 | 229 |
| 21 | OAR21_26542185 | 23719561 | 13.54455297 | 127 | 143 | 297 |
| 21 | OAR21_26609482 | 23771834 | 17.31559925 | 275 | 42 | 250 |
| 21 | s14192 | 24245811 | 16.04137848 | 261 | 52 | 254 |
| 21 | s25250 | 24416847 | 17.59267059 | 27 | 328 | 212 |
| 21 | OAR21_34167867 | 30592661 | 14.80420426 | 69 | 242 | 249 |
| 21 | s25105 | 45636271 | 15.93116344 | 16 | 359 | 192 |
| 21 | OAR21_54114533 | 48838644 | 16.29938373 | 37 | 287 | 236 |
| 22 | s02457 | 8336952 | 13.9829881 | 15 | 413 | 139 |
| 22 | OAR22_10980712_X | 9189837 | 16.10216102 | 282 | 39 | 240 |
| 22 | OAR22_12868251 | 10927246 | 13.80997331 | 4 | 450 | 113 |
| 22 | OAR22_12886314 | 10944905 | 13.55117098 | 446 | 5 | 116 |
| 22 | OAR22_38946909 | 34121320 | 17.15680873 | 422 | 10 | 135 |
| 22 | OAR22_40839521 | 36097157 | 17.60449111 | 23 | 358 | 186 |
| 22 | s27788 | 37127060 | 15.08004031 | 170 | 109 | 286 |
| 22 | s25057 | 39433385 | 14.12381379 | 235 | 92 | 239 |
| 22 | OAR22_45911188 | 40874829 | 14.20776481 | 80 | 221 | 265 |
| 22 | OAR22_46305001 | 41259547 | 13.61297655 | 63 | 283 | 220 |
| 22 | s50123 | 42997377 | 15.55969371 | 213 | 82 | 272 |
| 22 | OAR22_49529205 | 44176943 | 13.56685058 | 270 | 49 | 247 |
| 22 | s58936 | 44247327 | 15.44857656 | 248 | 65 | 254 |
| 23 | s41088 | 2357408 | 14.68155492 | 289 | 37 | 241 |
| 23 | OAR23_6952059 | 6296614 | 14.08626229 | 79 | 213 | 275 |
| 23 | OAR23_19521373 | 18404296 | 14.49128302 | 142 | 142 | 277 |
| 23 | s57038 | 22105490 | 20.58797892 | 116 | 163 | 288 |
| 23 | s18696 | 23048389 | 15.47301806 | 122 | 135 | 262 |
| 23 | OAR23_27658605 | 26522834 | 14.3792066 | 270 | 46 | 245 |
| 23 | OAR23_31734596 | 30350528 | 14.58231944 | 186 | 95 | 285 |
| 23 | OAR23_33227623 | 31541493 | 17.14585398 | 11 | 423 | 130 |
| 23 | OAR23_35510473 | 33556377 | 23.48392404 | 53 | 254 | 260 |
| 23 | OAR23_38365072 | 36293219 | 17.75615256 | 265 | 53 | 249 |
| 23 | OAR23_39546165 | 37298993 | 14.98781283 | 359 | 25 | 183 |
| 23 | OAR23_39944789 | 37693255 | 14.50255032 | 185 | 102 | 280 |
| 23 | OAR23_39971449 | 37717812 | 16.22130813 | 260 | 56 | 250 |
| 23 | OAR23_40003309 | 37749930 | 15.61834002 | 158 | 126 | 283 |
| 23 | s45885 | 39069594 | 14.37612773 | 424 | 8 | 134 |
| 23 | s32452 | 39599690 | 15.60362607 | 72 | 262 | 232 |
| 23 | OAR23_42278026 | 39890079 | 16.35968591 | 362 | 18 | 185 |
| 23 | s20449 | 39909234 | 13.66311933 | 22 | 360 | 184 |
| 23 | OAR23_57454082 | 53935751 | 18.13730546 | 210 | 71 | 285 |
| 24 | s62308 | 4435644 | 14.52266849 | 110 | 165 | 279 |
| 24 | OAR24_23777790 | 22021018 | 15.04645111 | 436 | 8 | 122 |
| 24 | s04085 | 22769125 | 15.48328521 | 183 | 97 | 282 |
| 24 | OAR24_32895087 | 30083398 | 13.63820012 | 10 | 436 | 121 |
| 24 | s30694 | 32349389 | 14.53109959 | 6 | 438 | 123 |
| 24 | s01323 | 39101148 | 13.60339987 | 343 | 30 | 192 |
| 24 | s55689 | 40717526 | 16.37889479 | 333 | 34 | 200 |
| 25 | s49400 | 187760 | 16.54123529 | 44 | 281 | 242 |
| 25 | s66893 | 368575 | 17.18228999 | 353 | 17 | 197 |
| 25 | s15407 | 591969 | 13.65697915 | 82 | 196 | 281 |
| 25 | OAR25_7921364 | 8097890 | 15.73490635 | 125 | 159 | 283 |
| 25 | OAR25_9378394 | 9410099 | 18.26342678 | 3 | 457 | 106 |
| 25 | s73544 | 9616653 | 16.4571125 | 368 | 21 | 177 |
| 25 | OAR25_16816914 | 16259639 | 13.89788305 | 269 | 53 | 241 |
| 25 | s37347 | 17904090 | 16.00111954 | 178 | 115 | 273 |
| 25 | OAR25_21471714 | 20750339 | 13.97325994 | 317 | 26 | 223 |
| 25 | OAR25_25242998 | 24185116 | 14.70737778 | 240 | 74 | 253 |
| 25 | s59508 | 26747002 | 13.50304427 | 30 | 332 | 204 |
| 25 | OAR25_27992869 | 26769599 | 14.69197403 | 93 | 188 | 286 |
| 25 | s24247 | 27616367 | 15.696256 | 286 | 51 | 228 |
| 25 | OAR25_29454744 | 28120276 | 16.70255071 | 64 | 245 | 219 |
| 25 | OAR25_29853310 | 28534443 | 13.79133797 | 4 | 480 | 83 |
| 25 | OAR25_30372586 | 29046746 | 22.10657722 | 211 | 90 | 266 |
| 25 | OAR25_31163834 | 29829111 | 14.80392865 | 21 | 400 | 145 |
| 25 | s25673 | 31246866 | 16.15565115 | 41 | 287 | 239 |
| 25 | OAR25_37809953 | 36015994 | 13.52668123 | 193 | 98 | 246 |
| 25 | s36865 | 36788528 | 15.02316017 | 240 | 74 | 248 |
| 25 | s16017 | 40941950 | 13.64717647 | 210 | 69 | 288 |
| 26 | s37144 | 1773622 | 14.52122384 | 478 | 3 | 85 |
| 26 | OAR26_6237925 | 5550505 | 17.16986839 | 54 | 272 | 241 |
| 26 | OAR26_6305965 | 5618975 | 22.50274708 | 21 | 373 | 173 |
| 26 | OAR26_8271562 | 6140010 | 18.11825128 | 4 | 451 | 111 |
| 26 | OAR26_8526649 | 6390471 | 13.64272971 | 4 | 481 | 80 |
| 26 | OAR26_9556680 | 7398653 | 14.87254665 | 6 | 461 | 100 |
| 26 | OAR26_9742089 | 7576285 | 15.94573185 | 33 | 314 | 220 |
| 26 | OAR26_14922456 | 12157701 | 15.41953258 | 408 | 16 | 143 |
| 26 | s59738 | 13117851 | 14.5052449 | 159 | 118 | 288 |
| 26 | s66089 | 13270083 | 13.87443257 | 27 | 333 | 205 |
| 26 | OAR26_17518698 | 14644438 | 13.53655403 | 368 | 14 | 169 |
| 26 | OAR26_29996287 | 25917946 | 13.7529124 | 286 | 45 | 236 |
| 26 | OAR26_34945353 | 30625379 | 13.67757448 | 199 | 89 | 279 |
| 26 | s68466 | 35198098 | 14.20839468 | 154 | 126 | 287 |
| 26 | s04492 | 35994904 | 14.05294906 | 191 | 93 | 281 |
| 26 | s50736 | 43029260 | 14.2686986 | 373 | 16 | 178 |
| 27 | s05800 | 6494952 | 14.79748948 | 10 | 445 | 72 |
| 27 | OARX_14861261 | 8957306 | 14.62511078 | 184 | 232 | 146 |
| 27 | OARX_48129885 | 41560832 | 14.74936618 | 75 | 393 | 86 |
| 27 | OARX_55032299 | 48942926 | 15.19931528 | 296 | 102 | 128 |
| 27 | OARX_106780979 | 86909468 | 15.10950837 | 122 | 280 | 150 |
| 27 | OARX_109922971 | 90782534 | 14.03660219 | 131 | 270 | 158 |
| 27 | OARX_110184293_X | 90973799 | 15.01453961 | 38 | 461 | 66 |
| 27 | s10353 | 91339403 | 14.3856002 | 59 | 443 | 64 |
| 27 | s71449 | 91457111 | 16.07997836 | 297 | 137 | 126 |
| 27 | OARX_111349974 | 92085118 | 13.59141341 | 114 | 343 | 106 |
| 27 | OARX_124978161 | 100593817 | 16.18418605 | 273 | 126 | 138 |
| 27 | s20530 | 127251155 | 13.91036321 | 139 | 300 | 121 |
